# Simple Quantitative PCR Approach to Reveal Naturally Occurring and Mutation-Induced Repetitive Sequence Variation on the *Drosophila Y* Chromosome

**DOI:** 10.1371/journal.pone.0109906

**Published:** 2014-10-06

**Authors:** John C. Aldrich, Keith A. Maggert

**Affiliations:** Department of Biology, Texas A&M University, College Station, Texas, United States of America; North Carolina State University, United States of America

## Abstract

Heterochromatin is a significant component of the human genome and the genomes of most model organisms. Although heterochromatin is thought to be largely non-coding, it is clear that it plays an important role in chromosome structure and gene regulation. Despite a growing awareness of its functional significance, the repetitive sequences underlying some heterochromatin remain relatively uncharacterized. We have developed a real-time quantitative PCR-based method for quantifying simple repetitive satellite sequences and have used this technique to characterize the heterochromatic *Y* chromosome of *Drosophila melanogaster*. In this report, we validate the approach, identify previously unknown satellite sequence copy number polymorphisms in *Y* chromosomes from different geographic sources, and show that a defect in heterochromatin formation can induce similar copy number polymorphisms in a laboratory strain. These findings provide a simple method to investigate the dynamic nature of repetitive sequences and characterize conditions which might give rise to long-lasting alterations in DNA sequence.

## Introduction

A significant portion of most eukaryotic genomes is composed of repetitive DNA elements [1]. It is estimated that as much as 1/3 of the genome of *Drosophila melanogaster* is composed of such sequences [2], [3]. This fraction is largely confined to centric and telomeric regions where it forms constitutive heterochromatin, cytologically distinct in its appearance and genetically distinct in its properties. Constitutive heterochromatic sequences are largely of two types: middle repetitive sequences such as transposable elements, and highly repetitive major- and micro-satellite sequences [3]–[8]. Although highly-repetitive heterochromatic satellite sequences (*e.g.*, AAGAG, AATAT, AAGAGAG) house a variety of biological phenomena including centromere function, chromosome cohesion and pairing, nuclear organization, control of recombination, species-compatibilities, replication rate, and gene regulatory variation [9]–[14], understanding their function mechanistically has lagged far behind sophisticated understanding of the function of euchromatic sequences. This is due in large part to the difficulty in handling these sequences with modern molecular biological approaches. Next-generation sequencing technology has increased the rate with which we have learned about the structure and variation of euchromatin, but the heterochromatic portion of the genome remains relatively ignored in its characterization [3], [15], even very recently not rising to the level of notice in debate over the role of “junk”DNA [16], [17].

The *Y* chromosome of *Drosophila melanogaster* is a useful tool for understanding the evolution of satellite sequences and their contribution to genome regulation [18], [19]. The *Drosophila Y* chromosome is naturally variant, can be made supernumerary in males or females, is dispensable in males, has very few genes, is a component of numerous chromosome rearrangements, and its functional and sequence elements have been roughly mapped. Apart from genes necessary for male fertility and a small set of non-essential genes, the *Y* chromosome is almost entirely composed of repetitive DNA such as megabase-long blocks of satellite repeats –variously called alphoid repeats, alpha-heterochromatic repeats, satellite repeats, simple repeats, simple satellite repeats (SSRs), highly-repetitive DNAs, repetitious DNAs, *etc*. –as well as interspersed or clustered transposable elements, the repetitive Ribosomal RNA genes (*rDNA*), and other genetic elements [5], [6], [20]. *Y* chromosomes isolated from diverse populations affect a number of phenotypes including temperature sensitivity, sex ratio, heterochromatin formation, male fitness, innate immunity, and others [21]–[25] and may do so by differentially influencing genome-wide transcription. Although some of these effects can be attributed to *rDNA* copy number polymorphisms [26], [27], it is likely that the balance of unmapped variation lies within satellite sequence [28].

“Complex”euchromatin contains ample sequence variation to analyze for function, while the sequence variation of satellites has fewer parameters in which it can vary. Blocks of satellite repeats can vary in their length (*i.e.*, copy number), homogeneity (*i.e.*, polymorphisms in the consensus repeat unit), punctuation (*i.e.*, location, type, and copy number of transposable elements or transposable element remnants), orientation (*e.g.*, AAGAG or CTCTT in relation to the centromere), juxtapositions (*e.g.*, the types or arrangements of satellite repeats at junctions), or linkage (to specific chromosomal locations). There have been some attempts to explore these features, but it is difficult to apply standard molecular tools to understand the architecture of the heterochromatin. Currently, studies to address variation have chiefly measured linkage and copy number using fluorescence *in situ* hybridization or Southern blot analysis.

Acknowledging that no approach is perfect, and following on our recent experiments [29], [30] demonstrating the importance of *rDNA* copy number variation in heterochromatin formation and *Y*-linked Regulatory Variation, we wished to develop a similar method to quantify the copy number of satellite repeats that is (i) simple, (ii) robust, (iii) sensitive, (iv) quantifiable, (v) inexpensive, (vi) fast, and (vii) can be integrated with other approaches to provide an understanding of the arrangements of satellite DNAs.

“Real-Time”or “Quantitative” Polymerase Chain Reaction (qPCR) has been successfully used to accurately quantify *rDNA* copy-number variation in numerous studies [31]–[35], and is theoretically directly applicable to any repetitive sequence element whose repeat unit is longer than the typical approximately 100 base pair product of qPCR. The absence of unique primer binding sites in blocks of short (*e.g.*, pentameric or heptameric) satellites makes avoidance of primer-primer annealing the chief difficulty. An assay that circumvented this problem and allowed the amplification and quantification of simple telomeric repeats has been developed [36], [37]. We thought this assay could in principle be adapted for heterochromatic satellites, which in many regards pose the same problems as telomeric DNA: short, homogenous, high copy number. In this study, we show that we successfully adapted this qPCR technique for the quantification of pentameric satellites. We validated precision using a dilution series and *Y* chromosome aneuploids, and found that geographically diverse *Y* chromosomes harbor previously uncharacterized satellite copy-number polymorphisms. Furthermore, we applied the approach to discover that long-term exposure to a mutation affecting heterochromatin formation and genome stability, the *Su*(*var*)*205* locus which encodes the HP1a gene product, results in measurable changes in satellite copy number, suggesting that much like *rDNA* [38]–[40], satellite copy number stability is regulated by chromatin factors.

## Results

### Design of Real-Time-Based Quantitative PCR Approach

Large blocks of simple pentameric repeats AACAC and AAGAC are constituents of the *Drosophila Y* chromosome [7], [41], accounting for less than about 2% and about 20%, respectively, of the *Y*; the remaining balance largely resides in the pericentric heterochromatin of chromosome *2*. In order to investigate copy number variation of these repeats, we adapted a Real-Time Quantitative PCR (qPCR) assay, originally designed for quantifying telomeric repeat copy number by Cawthon [36], [37], that would allow us to quantify their relative copy number. The reaction used primers with designed self-incompatibilities to disfavor primer-dimer formation and instead heavily-favor template-dependent and product-dependent priming. The products of template-dependent synthesis created self-compatible products, which were preferentially amplified exponentially as is normal in PCR reactions.

Five design elements were incorporated into primer design. First, a “Forward” primer matching the repeat (*e.g.*, AACAC) contained a base-pair change (therefore a mismatch with the repeat) every 5 nucleotides. Second, the “Reverse” primer (*e.g.*, GTGTT) did so as well, but the mismatch was not the same as that on the “Forward” primer. Third, the primer set (Forward and Reverse) converged at a position in the repeat that was not complementary (*i.e.*, they did not overlap at their 3′ ends). Fourth, the primers each contained five nucleotides at their 5′ ends that were not homologous to the repeat. Fifth, the primers had nucleotides at their 3′ ends such that the best primer-primer annealing configurations had minimally two 3′ mismatches [37].

**Figure 1:**
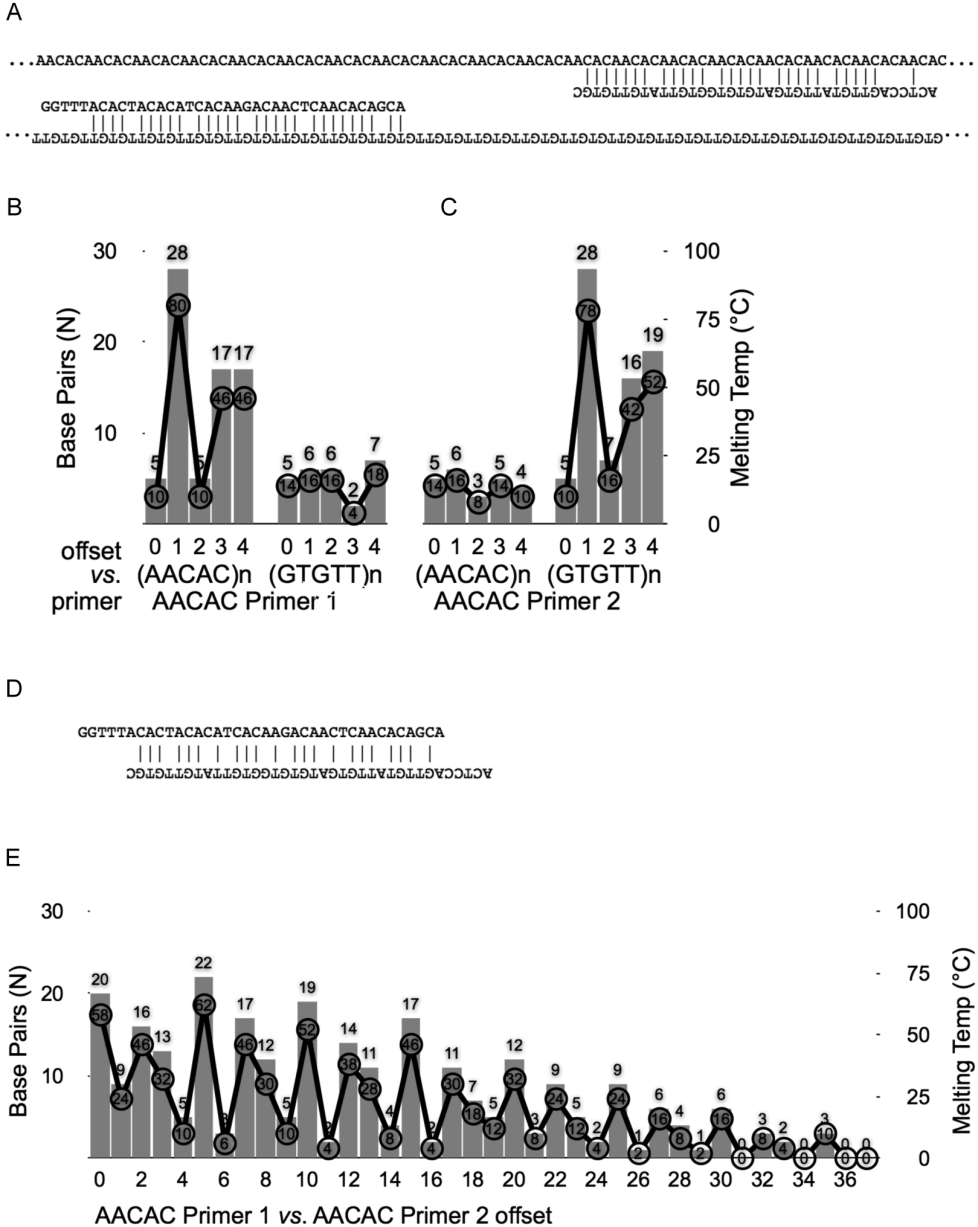
Computational validation of qPCR primers directed at AACAC satellite. (**A**) Representation of homogenous block of AACAC repeat denatured and annealing to AACAC Forward and AACAC Reverse. Vertical lines indicate base pairs. (**B**) AACAC Forward hypothetically annealing to each of the five “phases”of AACAC (*i.e.*, AACAC, ACACA, CACAA, ACAAC, and CAACA) and it’s reverse-complement. Bars indicate the number of base pairs possible in that phase, and line indicates *ad hoc* “melting” analysis. AACAC Forward has a strong preference for one phase and no affinity for the reverse-complement strand of the repeat. (**C**) as in (B), but with AACAC Reverse. (**D**) Shows the best possible pairing between AACAC Forward and Reverse. (**E**) AACAC Forward and AACAC Reverse pairing with every possible degree of overlap, from completely (at “0 offset”) to single 3′-most nucleotides pairing (at “37 offset”). Bars and lines are as in (B–C). In no case is AACAC Forward/Reverse dimer preferred over annealing to genomic targets (A–C) or the product of previous amplification.

This design balanced qPCR primers (i) effectively binding to and priming from the genomic satellite DNA repeat, (ii) exponentially amplifying from products of previous cycles of the “chain reaction” amplification, and (iii) avoiding primer-dimers forming between primers both directed at the same repetitious DNA sequence. Key to this end, introduced base pair mismatches (“First” and “Second” design elements above) were out of phase with each other and compromised the binding between primers and target genomic DNA, but more egregiously compromised binding with each other. This is clarified in Figure 1A, which shows the sequence of primers directed at AACAC repeats. A homogenous block of AACAC binds “AACAC Forward” (“sense,”homologous to AACAC) and “AACAC Reverse”(“antisense,”homologous to GTGTT), each with multiple single mismatches spread throughout the primer length.

### Computational Justification of Approach

Computational analyses of the separate AACAC Forward and AACAC Reverse primers show that the designs retain the preference for annealing to a defined phase of the pentanucleotide repeats (Figure 1B–C, and as shown in Figure 1A). In this case, AACAC Forward anneals best when the 5′end of the primer overlaps with the second nucleotide of AACAC (aAcac) and ends at the fourth nucleotide in the repeat unit (aacAc). Annealed primers in this phase pair perfectly with genomic sequence at 28 bases (Figure 1B, offset 1 *vs*. AACAC), including five clusters of five consecutive pairs. There is no simple method to accurately predict pairing energy or melting temperature of intentionally mismatched primers that can compete for multiple sequences, but using a thumb-rule (2°C for each A/T and 4°C for each G/C) is suggestive of the predilection for this phase of binding. By this calculation, the melting temperature is 80°C for this phase and less than 50°C for other phases. AACAC Reverse also has a preference for 5′ alignment with the second nucleotide of the pentanucleotide repeat (gTgtt) (Figure 1C, offset 1 *vs*. GTGTT), and also ends on the fourth nucleotide (gtgTt). Pairing in this phase has a similar distribution of five consecutive paired bases, and a similar thumb-rule melting temperature of 78°C.

Primer-dimers are a constant concern in primer design, and the repetitious nature of the target sequences makes avoidance difficult because there are multiple pairing arrangements that are a function of the repeat-length. We analyzed the number of possible base pairs forming given every degree of overlap between AACAC Forward and AACAC Reverse (Figure 1D–E). The repeat unit length is clear as a local maximum every five nucleotides, flanked by two far-sub-optimal arrangements around each local maximum (*i.e.*, offset by 4–5–6 nucleotides, 9–10–11, *etc*.). AACAC Forward and Reverse best pair with an offset of five nucleotides (as shown in Figure 1D) which creates eight internal mismatches, disrupts pairing of more than 3 consecutive bases, and leaves 3′ mismatches on both ends, which significantly inhibits polymerase elongation. This total number of base pairs (22/38) is lower than reactions primed on genomic DNA (28/38 for AACAC Forward or AACAC Reverse), and the thumb-rule approximate melting temperature (62°C) is less favored than either primer annealing to genomic DNA. Additionally, this “best” match allows only ten nascent nucleotides (both 5-base pair 3′-overhangs) to be incorporated during a PCR extension, much less signal than would included by even the worst priming of a previously-amplified primer (40 nucleotides if the primers annealed at juxtaposed genomic repeats; 38 for the primer plus the 2 of non-overlap in the repeat unit, …aacaCAacac…) or a valid genomic DNA-primed event. After the second successive cycle of priming and elongation, there are no longer any mismatches between primer and PCR-produced template, thus normal qPCR conditions are established.

### Experimental Validation of Quantitative PCR-Based Satellite Quantification

**Figure 2:**
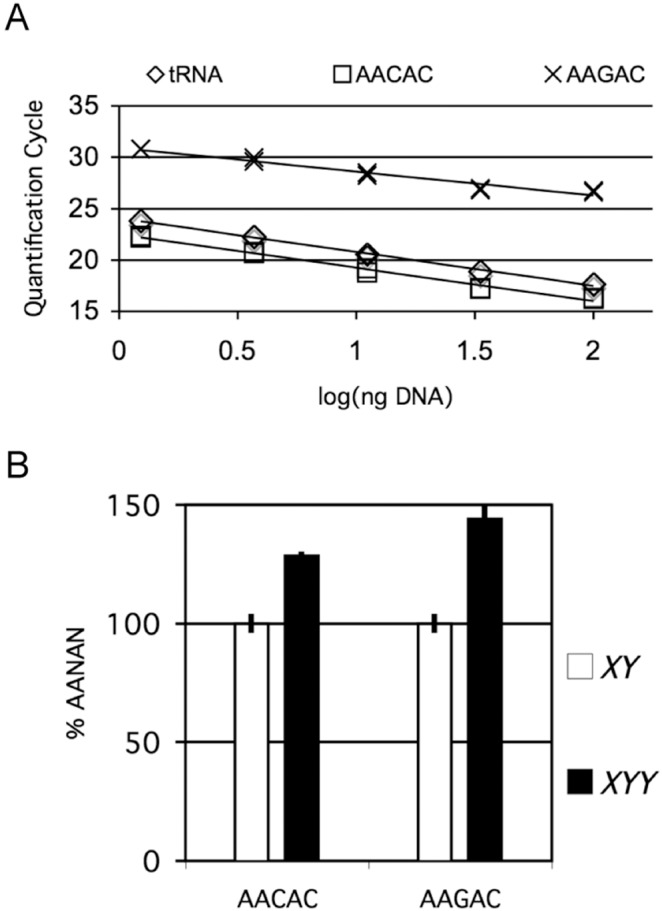
Experimental validation of qPCR assay to measure satellite copy number. (**A**) Quantification cycle (C_q_) of duplicate qPCR reactions plotted as a function of template DNA per reaction. X-axis represents log_10_ of an approximately 100-fold dilution series. (**B**) Quantification of satellite copy number in *X*/*Y* males and to *X*/*Y*/*Y* males, relative to *X*/*Y* (defined as 100%). Error bars represent standard error of the mean (S.E.M.) derived from triplicate reactions.

To confirm the robustness of our assay, we performed qPCR reactions on isolated genomic DNA over approximately a 100-fold dilution range (1.23 ng–100 ng per reaction) surrounding optimal conditions determined empirically in other studies ([31], Aldrich and Maggert, submitted). Over an intermediate range (3.7 ng–33.3 ng), we observed a very high correlation (R^2^ = 0.99) between template concentration and quantification cycle (C*_q_*, [42]) using primers directed at the copy number stable multicopy tRNA^K-CTT^ gene [31], AACAC, or AAGAC (Figure 2A), which matched our experience with amplification of the middle-repetitive *35S*/*45S* ribosomal RNA gene [31] and others’experience with simple telomeric repeats [36]. In practice to assure robustness, we routinely perform reactions using DNA concentrations falling within the middle of this range (about 4–10 nanograms). We recommend this concentration, however our results indicate that fluctuations in the DNA concentration due to variation in extraction or errors in preparation will have negligible influence over the result.

We analyzed the raw data from the qPCR reaction of each target for efficiency using the LinRegPCR software package [43]. This analysis ascertains closeness-to-doubling (efficiency) with each qPCR cycle, thus a score of 2.0 is theoretically ideal. The efficiency values for tRNA^K-CTT^, AACAC, AAGAC, and AAGAG, respectively, are 1.86±0.003, 1.87±0.004, 1.87±0.002, and 1.86±0.004 (each calculated from 12 reactions, errors are standard errors of the mean). Although these values are below theoretical maximal efficiency, they are all similar, thus any correction that would be applied to the data to account for sub-ideal efficiencies would be applied equally to all values and are effectively canceled out when reporting relative values. These efficiency values are within generally accepted guidelines (90%–110%) despite the intentional mismatches in satellite-directed primer sets. *Post-hoc* melt-curve analysis confirmed that only single melting peaks were observed from these reactions, indicating single PCR products were amplified during qPCR (Figure S1), supporting our computational justification.

We next used the ΔΔC*_q_* method of analyzing qPCR results to quantify repeat copy-number of AACAC and AAGAC relative to that of the tRNA^K-CTT^ gene [31]. Although these satellite repeats have been cytologically mapped, little information about their overall abundance in the genome is available. They are found on the *Y* chromosome, which can be removed or made supernumerary without defects in viability, allowing us to manipulate *Y* chromosome copy number to monitor the sensitivity of our assay. We collected infrequent (frequency≈10^−4^) spontaneous primary nondisjunctional exceptional progeny from a *yellow^1^ white^67c23^*/*Y, 10B y* ^+^ stock [44], or created secondary nondisjunctional progeny (see Materials and Methods). Yellow + females were crossed to euploid brothers and *y* ^1^ *w* ^67c23^/*Y, 10B y* ^+^/*Y, 10B y* ^+^ progeny were identified by their duskier bodies, a consequence of the *Y*-terminal duplication of the *yellow* ^+^ gene translocation. We determined copy number of pentameric AACAC and AAGAC in sibling *X*/*Y* and *X*/*Y*/*Y* males (Figure 2B); data are shown as %AANAN (indicating either AACAC or AAGAC) with the values for *y* ^1^ *w* ^67c23^/*Y, 10B y* ^+^ (our reference chromosomes) defined as 100%. AACAC and AAGAC are thus treated separately because we cannot support an *a priori* expectation that the AACAC and AAGAC primers sets should prime qPCR reactions with the same metrics (annealing temperature, elongation rate, fluorescence, efficiency, *etc*.). Similarly, determining absolute copy number (using known tRNA copy number as a multiplier) is not valid.

As expected, males with an extra *Y* chromosome possessed elevated *Y*-linked AACAC and AAGAC repeats. By pooling siblings during DNA extraction, we lost data on standard deviations between individuals, hence the error bars report standard errors of the means. Based on these averages, we estimate that *Y*-linked blocks of AACAC and AAGAC contribute approximately 29% and 44% to the total amounts of those respective satellites to the euploid *y^1^ w^67c23^/Y, 10B y* ^+^ genome.

### Quantitative PCR Satellite Analysis Reveals Strain Differences in Satellite Repeat Copy Number

It is of note that our estimate of *Y*-linked AAGAC levels differs from a previously published estimate of 69% [7]. While this discrepancy might simply reflect the differing sensitivities of qPCR and radiolabelled or fluorescence *in situ* hybridization, it might also represent variation between different laboratory stocks. Repetitive sequence variation is of course not without precedence [28]. Examples include the expansion and contraction of ribosomal DNA in yeast and flies [39], [45], as well as interspersed satellite copy-number polymorphisms in humans and plants [46], [47]. Indeed, it is hypothesized that such variation may underlie the differential gene-regulatory effects of geographically divergent *Drosophila Y* chromosomes [23]–[25], [27].

**Figure 3:**
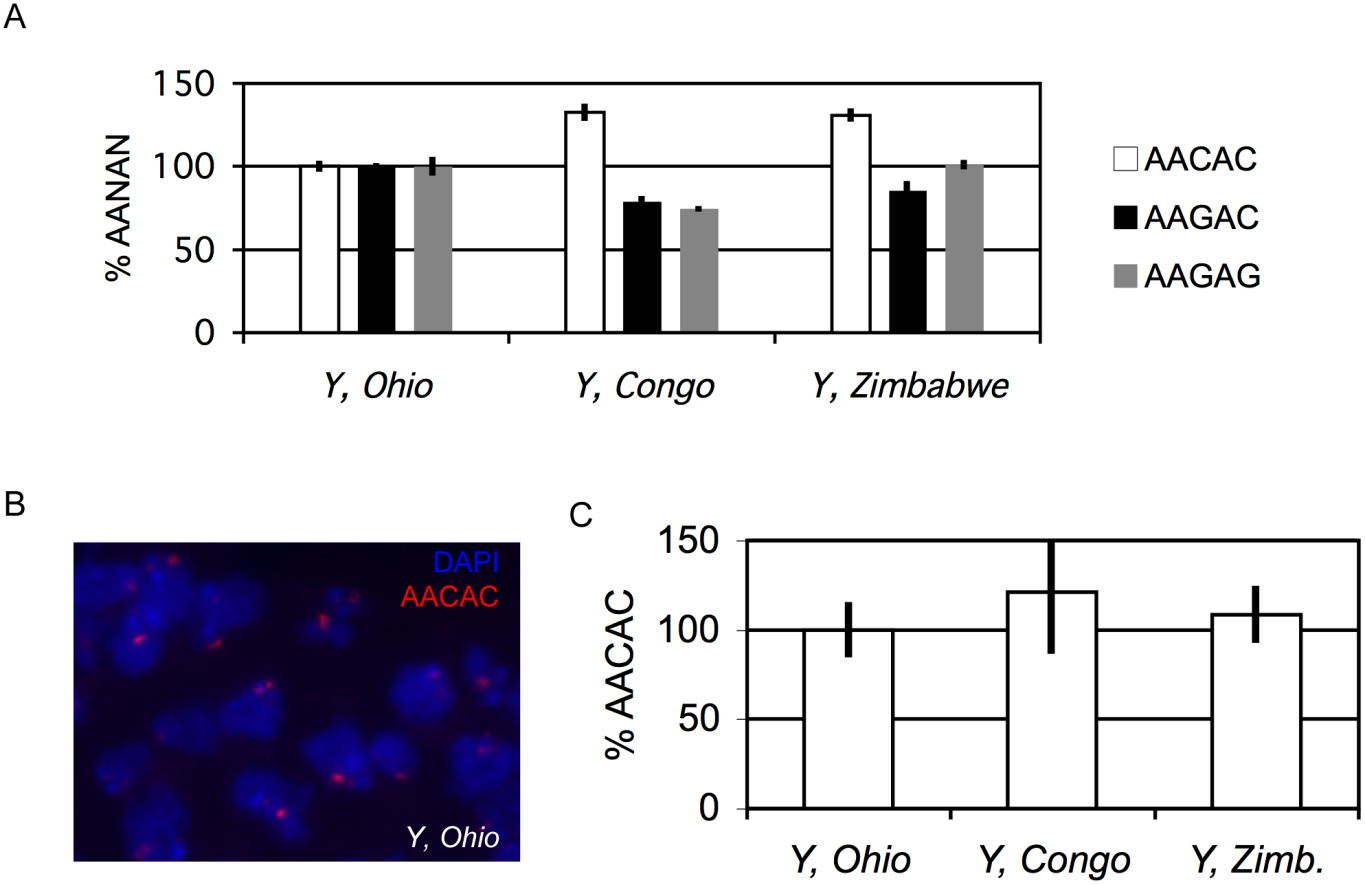
Quantification of *Y*-linked satellite copy number variation in geographically divergent lines. DNA and tissue samples obtained from males bearing *Y* chromosomes originally isolated from wild-caught flies. The genetic background of these males was otherwise isogenic. (**A**) Relative quantification of satellite copy number using qPCR. Percentages are relative to *Y*, *Ohio* (defined as 100%). Error bars represent standard error of the mean (S.E.M.) of triplicate qPCR reactions. (**B**) Fluorescence *in situ* hybridization to detect AACAC repeats (red) in squashed neuroblast cells derived from *Y*, *Ohio* larvae. DAPI stains DNA blue. (**C**) Quantification of *in situ* hybridization signals. Percentages calculated relative to *Y*, *Ohio* (defined as 100%). Error bars represent standard deviation (S.D.) of nuclei from thirty neuroblasts from each of three separate preparations per genotype (N = 90).

To address this possibility, we asked if we could detect satellite copy number differences on three of the *Y* chromosomes used in studies of unidentified *Y*-linked regulatory variation, referred to as *Y, Ohio*, *Y, Congo*, and *Y, Zimbabwe*. We introduced each of these *Y* chromosomes into otherwise-isogenic backgrounds by multiple patrilineal backcross to strains bearing homozygous recessive mutations on the *X* and autosomes [19], effectively replacing all non-*Y* nuclear and cytoplasmic DNA (so are thus *y* ^1^/*Y*; *bw* ^1^; *e* ^1^; *ci* ^1^ *ey* ^1^). In this way, we ensured that any observed satellite copy number differences were linked to the *Y* chromosome. Compared to *Y, Ohio* (our reference genotype for this experiment), AACAC levels were significantly higher (∼130%) on both *Y, Congo* and *Y, Zimbabwe* (P = 0.033 and 0.008, respectively, using Student’s t-test), while *Y, Congo* possessed relatively fewer copies of both AAGAC (∼79%) and AAGAG (∼75%) (P = 0.038 and 0.037, respectively). No significant difference was observed in *Y, Zimbabwe* AAGAC or AAGAG copy numbers compared to *Y, Ohio* (p = 0.098 and 0.862, respectively) (Figure 3A).

To support these findings and compare our approach to alternative techniques, we used fluorescence *in situ* hybridization to detect AACAC sequences in larval neuroblast nuclei (Figure 3B). Integration of data from ninety nuclei (thirty nuclei each from three separate brains dissected from sibling males) were largely consistent with our qPCR results: we confirmed significantly more AACAC in *Y, Congo* and *Y, Zimbabwe* compared to *Y, Ohio* (p = 0.008 and 0.036). The error bars in Figure 3C report standard deviation of integrated fluorescence from each nucleus and highlight the difficulty in quantification using fluorescence hybridization, which is prone to vagaries in hybridization, photobleaching, and chromosome spread quality. It is therefore impossible to say whether the differences in intensity are due to differences in cell-specific loss of AACAC copies or due to errors introduced during the procedure.

### Mutations Can Alter Repetitive DNA Copy Number

Several models exist to explain repetitive sequence copy number variation of the type that we see in wild-caught *Y* chromosomes. Polymerase slippage during replication is thought to be responsible for the changes in of simple sequence tracts while interchromosomal and intrachromosomal recombination events account for the gain or loss of larger portions of repetitive sequence [48]–[50]. Aberrant recombination in particular may be a common mechanism linking copy number variation to the type of genomic instability observed at other repetitive arrays [51]. In *Drosophila*, *rDNA* stability is regulated by a variety of chromatin factors (*e.g*. Histone H3 Lysine-9 methyltransferase, HP1a, DCR-2, CTCF) [26], [40], [52]. Removal of these factors by mutation results in genomic instability, increased damage, and repair defects in heterochromatin and copy number changes [40], [52], [53].

**Figure 4:**
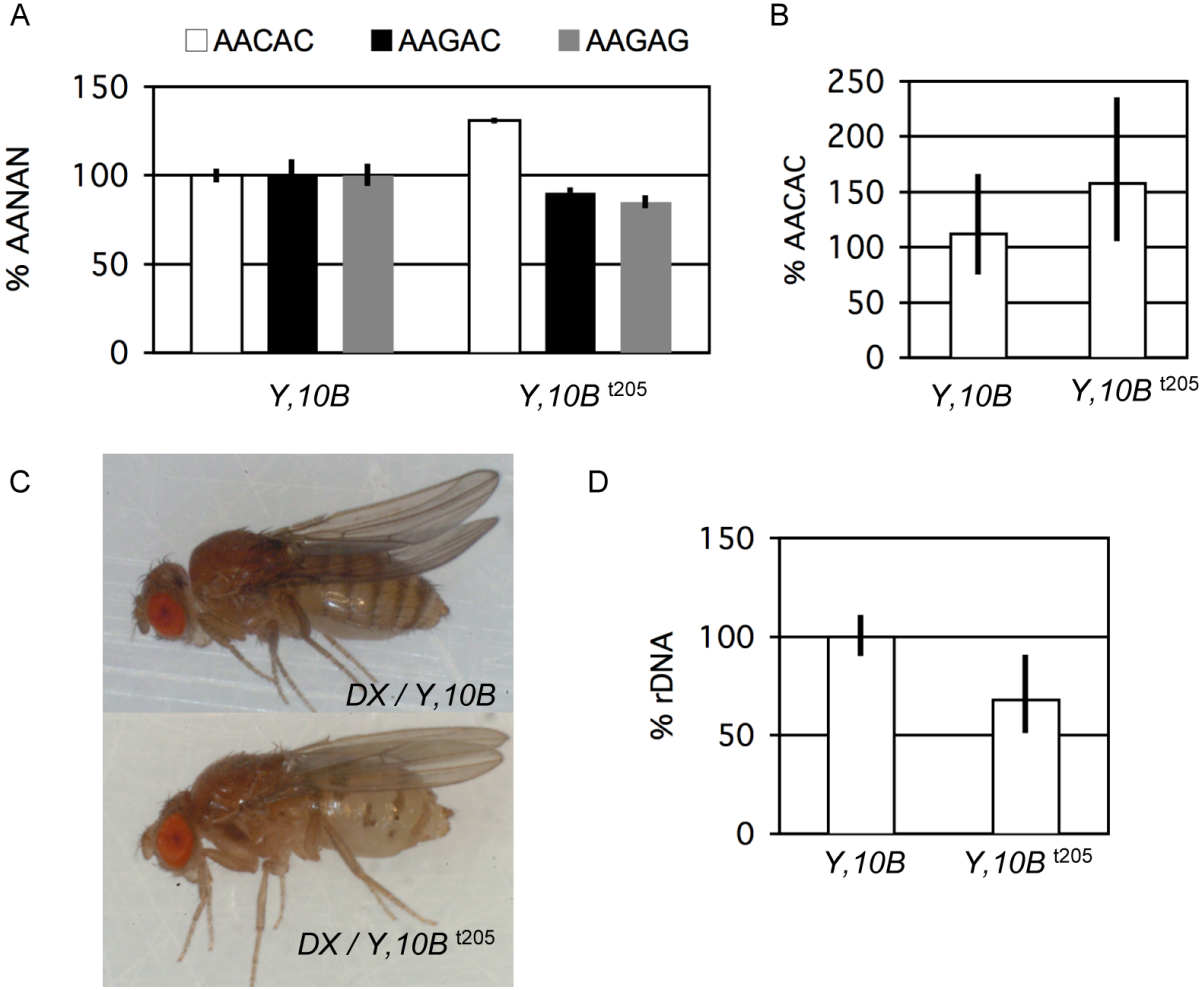
Quantification of satellite copy number variation after a exposure to *Su*(*var*)*205* ^05^/+ mutation. (**A**) Relative satellite copy number on *Y*, *10B* ^t205^ compared to *Y*, *10B* (defined as 100%). The chromosomes are originally from a single progenitor, but the former was maintained for 6 years in a *Su*(*var*)*205* ^5^/*CyO* mutant background. Error bars represent standard error of the mean (S.E.M.) of quadruplicate qPCR reactions. (**B**) DNA from four individual males bearing *Y*, *10B* ^t205^ were separately prepared and used as template for qPCR. Graphs show population average, error bars represent standard deviation (S.D.) of individuals within populations pooled with standard error of the mean (S.E.M.) of replicate reactions. Average AACAC copy number (in (A)) are comparable to the average of the population (in (B)), and population distributions remain detectably different (10B *vs*. 10B^t205^ in (B), P = 0.0336). (**C**) Images of female flies of genotype *C*(*1*)*DX*/*Y*, *10B* (top) and *C*(*1*)*DX*/*Y*, *10B* ^t205^ (bottom). The fly with *10B* ^t205^ as sole source of *rDNA* exhibits a strong bobbed phenotype, indicating significant *rDNA* loss. (**D**) qPCR determination of *rDNA* copy number in the flies from (C). Error bars report standard error of the mean of replicate qPCR reactions from pooled siblings.

To determine if mutations that alter heterochromatin-induced position effect variegation, *rDNA* expression, and *rDNA* stability also affect other satellite DNA copy numbers, we exposed our standard *Y* chromosome (*Y, 10B y* ^+^) to a mutation hypothesized to destabilize heterochromatic repeats. The *Su*(*var*)*205* gene encodes Heterochromatin Protein 1a (HP1a), which is enriched at sites of heterochromatin and is required for heterochromatic silencing [54], [55]. Notably, it is also required to maintain genomic stability in heterochromatin, and is involved in DNA repair of those sites [40], [56]. Given these properties, we hypothesized that the *Su*(*var*)*205* mutation might act dominantly and induce satellite DNA copy number changes on *Y, 10B y* ^+^. We placed a *Y, 10B y* ^+^ into a *Su*(*var*)*205* ^05^/+ mutant background and maintained it without selection for approximately 150 generations (approximately 6 years). In parallel we maintained a control *Y, 10B y* ^+^ in a wild-type *y* ^1^ *w* ^67c23^ background. After this, we moved the control *Y, 10B y* ^+^ and the six-year *Su*(*var*)*205* “tempered” counterpart (*Y, 10B* ^t205^) into the same isogenic background as above (*y* ^1^; *bw* ^1^; *e* ^1^; *ci* ^1^ *ey* ^1^) and quantified satellite copy number of AACAC, AAGAC, and AAGAG. We discovered that *Y, 10B* ^t205^ had ∼31% more AACAC compared to *Y, 10B* (p = 0.007) and apparent but non-significant decreases in AAGAC and AAGAG (p = 0.300 and 0.168, respectively) (Figure 4A).

To address the difference between individual chromosomes tempered by the *Su*(*var*)*205* mutation, we isolated DNA from four individual *y* ^1^/*Y, 10B* ^t205^; *bw* ^1^; *e* ^1^; *ci* ^1^ *ey* ^1^ males and performed qPCR for AACAC quantification on each. In each case, standard errors of the mean for replicate AACAC and tRNA reactions were pooled along with standard deviation calculations, and averages and pooled standard deviations of the four individuals are shown in Figure 4B. The variance around the mean is higher than when using pooled populations of flies (Figure 4A), however the averages of both *Y, 10B* and *Y, 10B* ^t205^ are comparable whether using pools (Figure 4A) or individuals (Figure 4B), and in both cases the difference in AACAC copy number between *Y, 10B* and *Y, 10B* ^t205^ is statistically robust (Figure 4B, P = 0.0036).

Peng and Karpen have previously observed that mutations in *Su*(*var*)*205* destabilize the *rDNA* [40], and they, Greil and Ahmad [33], and we [26] have shown that *Drosophila* strains with mutations in the methyltransferase responsible for creating the histone modification to which HP1a binds (*Su*(*var*)*3–9*) have few *rDNA*. We therefore expected that in addition to destabilizing AACAC, and potentially AAGAC and AAGAG, *rDNA* copy number would be different between *Y, 10B y* ^+^ and *Y, 10B* ^t205^. Crossing these two chromosomes to females bearing a compound *X* chromosome devoid of *rDNA* (*C*(*1*)*DX*, *y* ^1^ *f* ^1^ *bb* ^0^) revealed that the latter expressed a bobbed phenotype of etched and herniated abdominal dorsal cuticle, the manifestation of reduced translational capacity from reduced *rDNA* copy number (Figure 4C). *rDNA* copy number quantification using qPCR confirmed a loss of *rDNA* in the *Y, 10B* ^t205^ chromosome (Figure 4D). Hence, exposure to *Su*(*var*)*205* mutation affects other repetitive DNAs of the *Y* chromosome.

## Discussion

A number of methods currently exist for determining the copy number of satellite DNAs –the repetitive simple sequences that comprise nearly half of most eukaryotic genomes. These methods include quantification using fluorescence *in situ* hybridization [57], hybridization blots [7], and next-generation sequencing [58]. Each has benefits and drawbacks, therefore none are ideal, but all are useful depending on the specific investigation and limitations. The Real-Time Quantitative PCR (qPCR) technique adapted for this study is simple in that it requires only routine DNA purification, two specially-designed satellite-specific primers, two “denominator” comparison primers, and is mathematically simple to calculate relative amplifications. With the growing awareness that repetitive satellite DNA in centric constitutive heterochromatin may be linked to ecological variation or disease proclivity, this technique fills a large and growing need. The approach we describe here is simple to perform, robust to fluctuations in DNA concentration or preparation, sensitive to small changes (we estimate ∼5% based on standard error) in satellite repeat copy number, very low-cost and rapid.

The total time from living organism to data is less than one day, making it rapid and useful for most purposes. The ability to perform analyses using as little as one nanogram of genomic DNA also allows independent assessment of satellite copy number in old samples, individuals, or dissected tissues, far below the useful detection limits of Southern blot analyses. The molecular nature allows satellite quantification even in cell types or organisms without established cytology. The rapidity, flexibility, and cost-effective nature of this assay makes it useful to a large number of investigators, even without resources for more expensive approaches (*e.g.*, next-generation sequencing).

The design of primers should be broadly amenable to any satellite repeat. Although we only validate it here for pentameric repeat satellites, design of the mismatches are expected to be easier as the repeat length increases. Provided some foreknowledge of the repeat identities, use of this technique will allow investigators to begin to investigate questions about natural variation in copy number, or mutation- or treatment- induced changes to satellite copy number. To that end, between-satellite comparisons are not valid, nor are determinations of absolute copy number, using this technique. This is evident from the different C_q_ values in Figure 2A, which we believe to be a function of the parameters of binding, priming, and elongation of different repeat sequences, or other factors that cannot be normalized across different primer or target sequences. However, between-organism comparisons of satellite copy numbers are valid, allowing investigators to determine if mutations or treatments results in copy number variability.

We used both natural ecological variation and mutant analyses to validate our approach. Using qPCR, we noted heretofore undiscovered variation in satellite copy number in natural populations from wild-caught *Y* chromosomes from three different geographical sources. These polymorphisms, and others like them, likely contribute to phenomena such as *Y*-linked Regulatory Variation or the ability of different chromosomes to variably suppress epigenetic heterochromatin-induced position effect variegation *in trans* [23]–[27].

We also discovered that a mutation in the *Su*(*var*)*205* gene, which encodes HP1a, results in satellite instability of a subset of repeat types. Previous cytological work showed that *Su*(*var*)*205* mutation, and a histone methyltransferase in the same chromatin modification pathway (*Su*(*var*)*3–9*) both act dominantly to cause nucleolar (*rDNA*) instability [26], [33], [40], [52]. Moreover, the amount of damage (judged by repair foci in interphase cells) suggested that the damage was more widespread than just the *rDNA* [52]. Since it had not been mapped, it was undetermined if damage induced by *Su*(*var*)*205* heterozygosity was limited to the soma or could affect germ cells, and thus be a source of satellite variability in natural populations.

We showed that a chromosome maintained long-term in a mutant of *Su*(*var*)*205* was induced to alter satellite copy number (Figure 4). This finding was striking because it shows that mutations thought to act “epigenetically” may also act by altering chromosome structures at places that have not yet been investigated. HP1 appears to bind to all cytological heterochromatin, so discovery that AACAC was increased in copy number while AAGAC and AAGAG were reduced was not predicted. We imagine three possible explanations. First, it is possible that HP1 acts to stabilize some satellite sequences while destabilizing others. A mechanism for the former is apparent from the role of HP1 in establishing a chromatin structure conducive to silencing. The latter, while it has no obvious mechanism, is nonetheless logically consistent with our observation. Second, it is possible that *Su*(*var*)*205* stabilizes all satellite sequences, but loss of destabilized sequence is not the only consequence of instability. For example, while destabilizing may frequently lead to loss of DNA through intrachromosomal or interchromosomal recombination or damage/repair, it may also lead to increases. Mechanistically, this could be from one segregation product of any interchromosomal recombination event, but additionally increases in copy number could be accomplished by replication-coupled polymerase slippage, rolling-circle replication, re-replication, or some unknown programmed event. Third, it is possible that only a subset of satellites are affected by mutation in *Su*(*var*)*205* (*e.g.*, the *rDNA*), but there exists communication between different types of satellite. For example, a decrease in *rDNA* copy number alters heterochromatin formation, which results in selective pressure to enlarge other heterochromatic components to compensate. This idea, unencumbered by data, imagines that different forms of satellite are in balance in the genome and perturbations of one type will cause new equilibria to be reestablished by expansion or contraction of other interacting types.

Our anecdotal experience has been that stocks of some mutations – *Su*(*var*)*205* and *Su*(*var*)*3–9* among others – become stronger in their abilities to suppress variegation (their eponymous phenotype) after being established. While others have noted this, it has been informally accepted to be by selection as a consequence of the small populations and conditions of fly stock maintenance, we can now suggest that these mutations may also (or instead) induce copy number changes to unlinked satellite sequences, which themselves permanently alter phenotypes. The extent and consequence of such changes are unknown, but with qPCR copy number determination they may now be pursued.

## Materials and Methods

### Fly husbandry and stocks

All crosses and stocks were maintained on standard cornmeal molasses media at 25°C. *X/Y/Y* males were generated by crossing spontaneously occurring *y^1^ w^67c23^*/*y^1^ w^67c23^/Y, 10B y^+^* or *y^1^ w^67c23^/y^1^ w^67c23^/Y, B^S^* female primary nondisjunctants to *y^1^ w^67c23^/Y, B^S^* or *y^1^ w^67c23/^Y, 10B y^+^* males, respectively. For the former, *y^1^ w^67c23^/y^1^ w^67c23^/Y, B^S^* virgins were crossed to *y^1^ w^67c23/^Y, 10B y^+^*, then *y^1^ w^67c23^*/*Y, 10B y^+^/Y, B^S^* male offspring backcrossed to *y^1^ w^67c23^* to create and maintain secondary nondisjunctional strains which produce large numbers of *X/Y/Y* males. *X*/*Y*/*Y* were distinguished from their *X*/*Y* siblings by the severity of the Bar-stone or yellow+ phenotypes. Geographically diverse *Y* chromosomes (*Y, Ohio, Y, Congo*, and *Y, Zimbabwe*) were obtained from Bernardo Lemos [23]. Chromosomes were placed in an isogenic background by crossing males to *y^1^; bw^1^; e^4^; ey^R^* females and backcrossing to the maternal genotype until all four recessive markers were made homozygous. *y^1^ w^67c23^/Dp(1;Y) y+, P{w = RSw}10B (Y, 10B y^+^)* is described in [59]. *Y*, *10B* ^t205^ was generated by maintaining *Y, 10B* in a *y^1^ w^67c23^*; *Su*(*var*)*205^05^/CyO* background for approximately six years. Prior to quantification, *Y, 10B* and *Y*, *10B* ^t205^ were placed in an isogenic *y^1^; bw^1^; e^4^; ey^R^* background as above.

### Quantitative Polymerase Chain Reaction

Quantitative PCR (qPCR) analysis was performed with 12 µL reactions as described in [31]. DNA was extracted from adult flies homogenized in pools of ten and quantified using a NanoDrop ND-1000. 10 nanograms was used for each reaction (except where indicated). DNA from individual flies did not perform well in our reactions; to circumvent this problem the reactions in Figure 4B used 0.1 ng template. qPCR was performed with a StepOne Real-time PCR system and Power SYBR Green reagents (Applied Biosciences). The following conditions were used for 40 cycles: 95°C for 3 s; 50°C for 15 s; 60°C for 30 s. Relative differences were calculated using the “ΔΔCT” method. Each data point represents an average obtained from three or four qPCR reactions. P-values were calculated from untransformed ΔCq values using Student’s t-test.

Satellites were amplified using primers designed according to [37]. AACAC: GGTTTACACTACACATCACAAGACAACTCAACACAGCA and ACTCCAGTTG- TATTGTGATGTGTGGTGTTATGTTGTGC; AAGAC: GGTTTTAGCCAAGAGAA-GACCAGACACGACAACACAAGACTA and ACTCCATCTTGCCTTGTTTTGTC-CTGTCTCGTCTTTTCTTGCCTTGTCTA; AAGAG: GGTTTTAGAAGTGAAGAT-AAGAGTAGAGATGAGAAGACAA and ACTCCATCTCTACTCTCTTGTCTTCA-CTTCTGTTCTCTT. The endogenous control, tRNA^K-CTT^, was amplified using CTAGCTCAGTCGGTAGAGCATGA and CCAACGTGGGGCTCGAAC. Primers were used at a concentration of 0.5 µM.

### Fluorescent *in situ* hybridization (FISH) and Microscopy

Fluorescence probe was made by end-labeling oligonucleotides of the respective satellite repeat (*e.g.*, AACACAACACAACACAACACAACACAACAC) with digoxigenin-conjugated dUTP, and visualized with a mouse anti-digoxigenin antibody conjugated to rhodamine. Dissections, tissue preparation, and hybridizations were performed as described by Larracuente and Ferree (submitted). DNA was counterstained with 1 ng/mL DAPI (MP Biomedicals). All images were obtained using a Zeiss Axioskop 2 epifluorescence microscope running AxioVision (v. 4.6.3.0) with a 20X objective (numerical aperature = 0.5). Sequential excitation was performed at 543 nm (for Rhodamine) and 405 nm (for DAPI).

## Supporting Information

Figure S1 **Melt Curve Analysis of qPCR Primer Sets.** First derivative with respect to temperature of Relative Fluorescence Units (RFU) through the indicated temperature range. Derivative was calculated by ΔY/ΔX for each temperature interval after maximal fluorescence was set at 100%. Single major peaks indicate monophasic melting, indicative of single qPCR products with relatively-homogenous melting profiles.
(TIFF)
