# Supplementary figures and images for: Simple Quantitative PCR Approach to Reveal Naturally Occurring and Mutation-Induced Repetitive Sequence Variation on the Drosophila Y Chromosome
Source: PLoS One. 2014 Oct 6;9(10):e109906. doi: 10.1371/journal.pone.0109906 (PMC4186871; doi:10.1371/journal.pone.0109906)

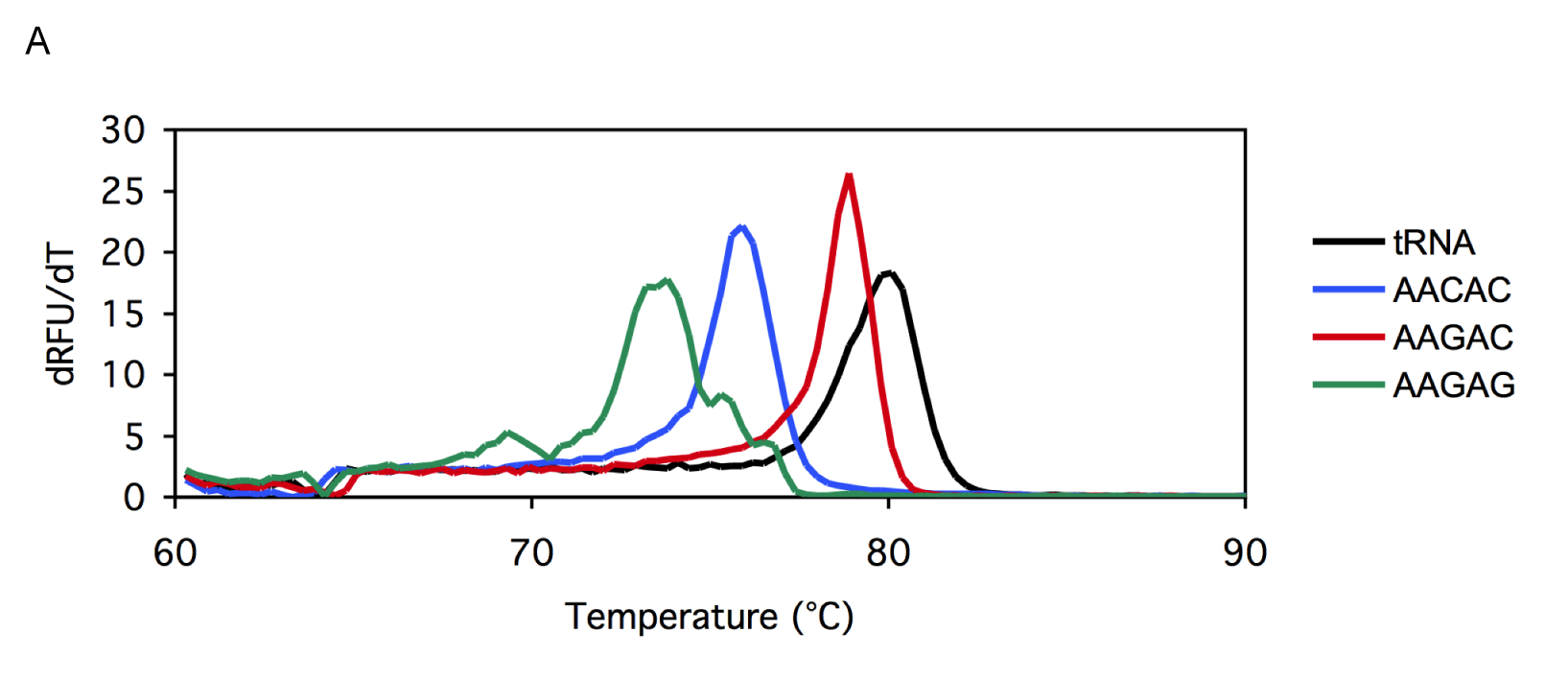

Supplement: Figure S1 — Melt Curve Analysis of qPCR Primer Sets. First derivative with respect to temperature of Relative Fluorescence Units (RFU) through the indicated temperature range. Derivative was calculated by ΔY/ΔX for each temperature interval after maximal fluorescence was set at 100%. Single major peaks indicate monophasic melting, indicative of single qPCR products with relatively-homogenous melting profiles. (TIFF) [file pone.0109906.s001.tiff]
